# Ring-Forming Polymerization toward Perfluorocyclobutyl and *Ortho*-Diynylarene-Derived Materials: From Synthesis to Practical Applications

**DOI:** 10.3390/ma14061486

**Published:** 2021-03-18

**Authors:** Eugene B. Caldona, Ernesto I. Borrego, Ketki E. Shelar, Karl M. Mukeba, Dennis W. Smith

**Affiliations:** Department of Chemistry and MSU Advanced Composites Institute, Mississippi State University, Mississippi State, MS 39762, USA; eib29@msstate.edu (E.I.B.); kes748@msstate.edu (K.E.S.); kmm1237@msstate.edu (K.M.M.); dsmith@chemistry.msstate.edu (D.W.S.J.)

**Keywords:** fluoropolymers, polymer synthesis, thermosets, high-performance polymers, cyclopolymerization, PFCB, BODA, enediynes, Bergman cyclization, carbonization

## Abstract

Many desirable characteristics of polymers arise from the method of polymerization and structural features of their repeat units, which typically are responsible for the polymer’s performance at the cost of processability. While linear alternatives are popular, polymers composed of cyclic repeat units across their backbones have generally been shown to exhibit higher optical transparency, lower water absorption, and higher glass transition temperatures. These specifically include polymers built with either substituted alicyclic structures or aromatic rings, or both. In this review article, we highlight two useful ring-forming polymer groups, perfluorocyclobutyl (PFCB) aryl ether polymers and *ortho*-diynylarene- (ODA) based thermosets, both demonstrating outstanding thermal stability, chemical resistance, mechanical integrity, and improved processability. Different synthetic routes (with emphasis on ring-forming polymerization) and properties for these polymers are discussed, followed by their relevant applications in a wide range of aspects.

## 1. Introduction

The design and development of polymers with beneficial and distinctive properties is critical for advanced technologies including nanomaterials, composites, photonics, microelectronics, and energy transformation [1,2,3,4,5]. In general, the properties of a given polymer can be predicted by the chemical features of its monomers and the polymerization process involved [6,7]. For instance, the preparation of polymers containing aromatic rings or alicyclic structures has attracted much attention because tremendous increase in chemical, mechanical, and thermal resistance is anticipated for such polymers [7,8]. The growing demand for high-performance polymers, particularly in the microelectronic, marine, and aerospace industries, has inspired the quest for aromatic and alicyclic polymers with intended applications as protective coatings, interlayer dielectrics, membrane materials, and optical components [9,10,11,12,13,14,15].

Fluoropolymers are classified as high-performance materials owing to their unique blend of complementary properties including chemical and thermal stability, mechanical durability, low surface energy, high insulation, and low refractive index [16]. These characteristics can all be linked to electronegative fluorine atoms and the strength of the resulting C–F and C–C bonds in fluoropolymers. However, many fluoropolymers, in general, are difficult to process through melting or use of a solvent, often leading to high manufacturing costs [16]. Thus, modified fluoropolymers (with ring or alicyclic structures in their repeat units) having reduced crystallinity are desirable [12]. Some commercial amorphous perfluoropolymers, Cytop^TM^ (perfluorovinyl ether cyclopolymer) and Teflon^TM^ AF (tetrafluoroethylene and perfluorodioxole copolymer) are leading examples.

**Scheme 1:**
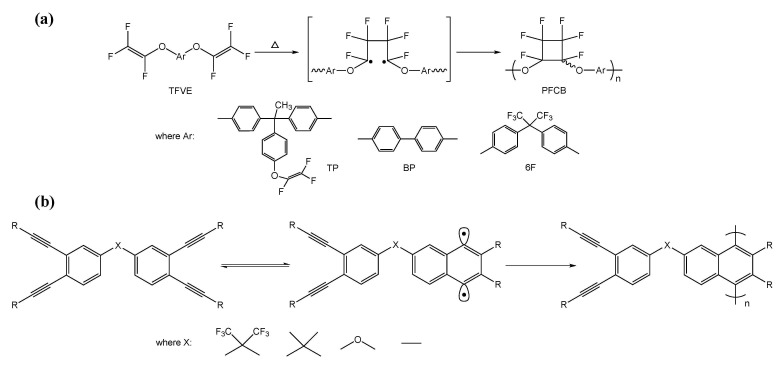
Synthesis of (**a**) PFCB polymer and (**b**) BODA monomer from TFVE monomer and bisphenol, respectively. Common aryl (Ar) and X groups are also shown. Variable R is typically H, phenyl, aliphatic, or heterocyclic groups.

First synthesized in the early 1990s by the Dow Chemical Co. (Midland, MI, USA) [17], perfluorocyclobutyl (PFCB) polyaryl ethers are a family of semi-fluorinated polymers that share several important properties with fully fluorinated polymers including chemical and moisture resistance, high thermal stability, and low dielectric constant, but with increased processability [11,12,16,18,19]. They are generally prepared by free radical mediated [2 + 2] thermal cyclopolymerization of functional aryl trifluorovinyl ether (TFVE) monomers at temperatures above 150 °C without using any catalysts or initiators (Scheme 1a) [12,18,19]. The cycloaddition of the fluoroolefins produces fluorinated cyclobutane rings, giving an overall amorphous PFCB polyaryl ethers with good solubility and solution processability for an array of new applications [9,11,12,20,21,22,23,24,25,26,27,28,29,30,31,32,33,34,35,36,37,38,39,40,41,42,43]. In the past decades, our group has been dedicated to the synthesis and applications of tailored PFCB polymers [12,20,44,45,46,47,48,49].

Polyarylenes are another family of high-performance polymers containing phenylene and/or fused aromatic rings as the main repeat unit [50,51,52,53]. They exhibit outstanding mechanical strength, chemical stability, optoelectronic properties, and their relatively high thermal stability makes them attractive precursors for the fabrication of electrically stable and conductive hard glassy carbon materials [50,53,54]. However, it is well-known that polymers containing rigid aromatic rings are fairly difficult to process, both by melting and in solution form [50,53]. Several approaches to preparing processable, hyperbranched polyarylenes by inclusion of flexible groups have been reported in the literature [55,56,57]. Our group has also reported the synthesis of bis-*ortho*-diynylarene (BODA) monomers from bisphenols (Scheme 1b) and their cycloaromatization (i.e., Bergman cyclization) and subsequent polymerization to a crosslinked network [50,52,54,58]. The branched structure of a BODA monomer, arising from its tetrafunctionality, provides excellent intermediate oligomer processability, allowing for easy thermal curing [50,53].

In this review, we aim to concisely highlight both PFCBs and ODA-based materials as two useful classes of aromatic and fluoroalicyclic polymers. Although these polymers have been widely discussed in the literature, the majority of the past and current reviews have focused broadly on the synthesis, properties, and industrial uses on each individual polymer [12,16,50,59]. Herein, we not only present in one review the synthetic routes toward production of new PFCB polymers (i.e., those containing large polycyclic aromatic hydrocarbons) and high carbon yielding ODA-based materials, but also emphasis on their preparation via ring formation, which leads to a remarkably unique combination of processability and performance properties. Ring-forming cyclopolymerization is attractive because it proceeds via undemanding polymerization conditions, requiring neither catalyst nor reagents, and produce little to no condensates over the course of the reaction. This naturally facile and high atom efficiency synthetic route can lead to promising mechanochemical and thermal reversibility properties, which are highly coveted for achieving stimulus-induced healing and other dynamic properties. The synthesis and properties of these polymers are first presented, followed by their use in fabricating high-performance materials for a wide range of industrial and engineering applications.

## 2. Synthesis of PFCB Polymers

Over the past decades, many researchers around the world have been focused on the preparation of PFCB polymers [27,31,32,33,44,45,46,47,48,49,60,61,62,63,64]. Monomers containing TFVE moieties have been developed and used to obtain PFCB polymers [31,60,61,62]. PFCB precursors bearing organic-inorganic cores including siloxanes and polyhedral oligomeric silsesquioxanes (POSS) have also been reported [28,65,66]. This section describes the use of various monomers and different methods for the preparation of PFCBs. The synthesis of the TFVE building blocks has been thoroughly expanded in many previous reviews [12,16,59] and will not be explored here.

### 2.1. PFCB-Based Copolymers and Blends

**Scheme 2:**
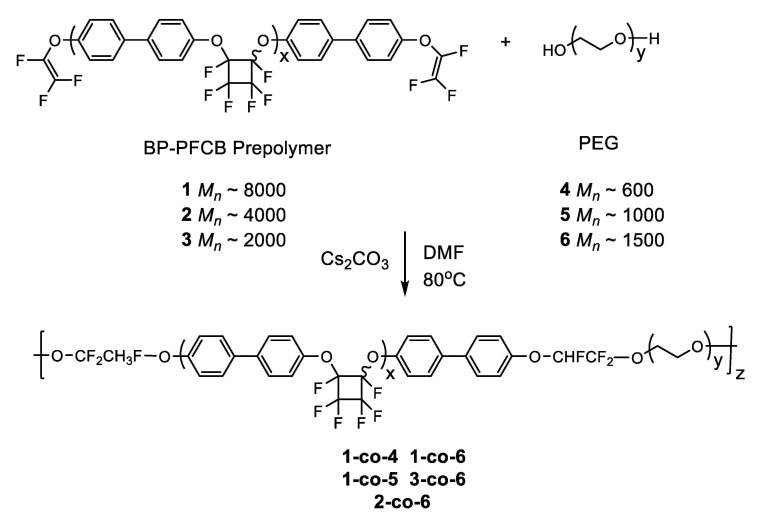
Synthesis of PEG/BP-PFCB block copolymers [44].

PFCB polymers are obtained by thermal [2 + 2] cyclopolymerization of TFVE-containing monomers (without using any catalysts or initiators) resulting in a four-membered perfluorocyclic ring. Our group reported, for the first time, the preparation of a series of amorphous PFCB multi-block copolymers through the formation of a fluorinated arylene vinylene ether (FAVE) bonded to a biphenyl (BP) prepared from poly(ethylene glycol) (PEG) and biphenyl-based (BP)-PFCB oligomers. The copolymers were synthesized via nucleophilic addition of hydroxyl end groups of PEG to TFVE functional groups (Scheme 2) [44]. Using NaH as base, PFCBs with unsaturated FAVE linkage rich with difluorovinylene (CF=CF) groups can be obtained. On the other hand, the utility of Cs_2_CO_3_ can result in PFCBs with stereorandom hydrofluoroethylene (CHFCF_2_) FAVE linkage [45]. To better understand the properties of the newly synthesized polymers, different wt% of hydroxytelechelic PEGs (4-6) and 4,4-bis(4-trifluorovinyloxy)biphenyl oligomers (1-3) were used during polymerization. The addition of higher number-average molecular weight (*M_n_*) monomers 5 or 6 to oligomer 1 showed an increase in *M_n_*, along with lower polydispersity of the resulting polymer. The decrease in polydispersity for 1-co-4, 1-co-5, and 1-co-6 was possibly due to the nucleophilic addition to the low molecular weight oligomer 1.

**Scheme 3:**
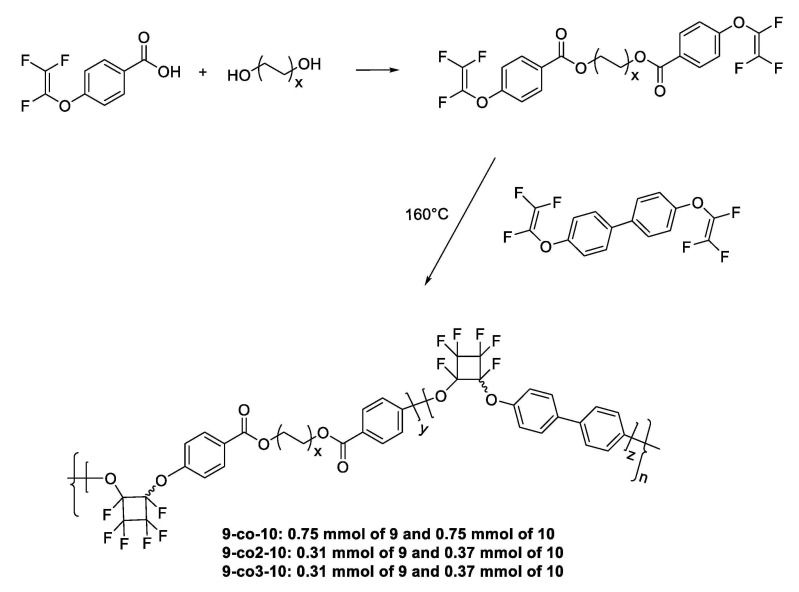
Copolymerization of TFVE-functionalized PEG with BP-PFCB [46].

The interest in polymer blend systems with minimum preparation steps for cost-effective processes and applications has rapidly increased over the last decades [44,46,67]. Polymer blend systems, which result in new materials with properties different from their parent components, are based on the physical combination of different polymers [44,67]. Although PFCB aryl ether polymers blended with fluorinated materials have been studied in the literature, less work on PFCB aryl ether blended with nonfluorinated monomers has been reported [44,46]. Brown et al. [46] reported the compatibilization of PEG with a biphenyl perfluorocyclobutyl (BP-PFCB) aryl ether polymers (Scheme 3). Monomer 9 was synthesized in a one-step fashion via esterification of 4-(trifluorovinyloxy)benzoic acid (7) with PEG (8). Monomers 9 and 10 were thermally copolymerized via step-growth [2 + 2] cycloaddition at 160 °C, affording the BP-PFCB copolymers. The PEG/BP-PFCB blends were prepared by dissolving PEG and BP-PFCB, both with *M_n_* ~20,000, in a 1:1 weight ratio containing 1–5 wt% compatibilizer 9-co2-10 in chloroform. Results showed that the addition of 9-co2-10 increases the compatibility of the two polymers and molecular adhesion, resulting in a polymer blend with low surface energy.

### 2.2. PFCB-Based Dielectric Polymers

**Scheme 4:**
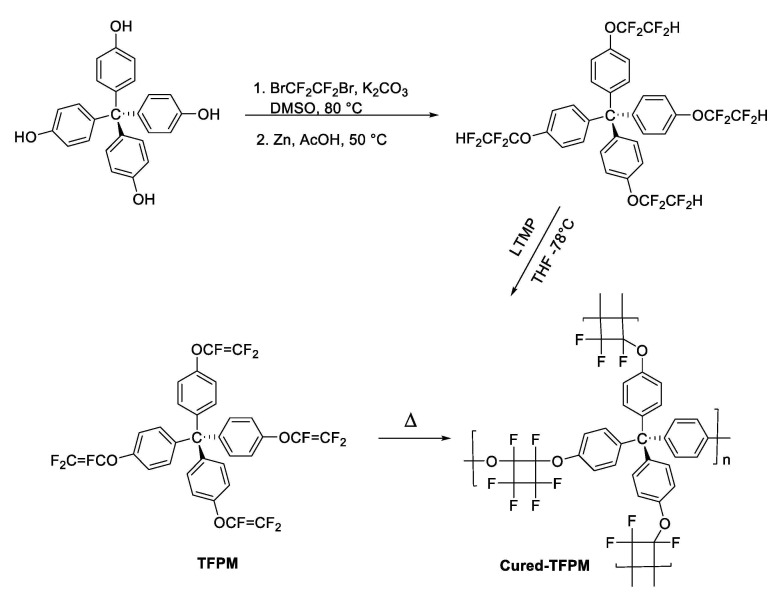
Preparation of TFPM and its cured form [30].

PFCB polymers with low dielectric constants (D_k_) and low dissipation factors (D_f_) have attracted much attention because of their potential use in the manufacture of high-performance electronic devices [28,29,30,31,33,68]. Although there are different ways of lowering the D_k_ and D_f_ values of polymeric materials, two of the most common techniques are (1) introducing less polarizable groups (e.g., C−H, C−C, C−Si, C−O, and C−F bonds) into the polymer backbone and (2) decreasing the material density by increasing the porosity [30,31,32]. Luo et al. [30] synthesized a new microporous PFCB aryl ether polymer from a tetrahedral structured monomer containing thermo-polymerizable TFVEs with −CF_2_H terminal groups (Scheme 4). Tetrakis(4-((1,2,2-trifluorovinyl)oxy)phenyl)methane (TFPM) monomer was synthesized via fluoroalkylation of 4,4,4″,4‴-methanetetrayltetraphenol, made unsaturated using lithium tetramethylpiperidine (LTMP), and thermally cured into a porous cross-linked polymer. The resulting PFCB polymer exhibited low D_k_ and D_f_ values of 2.36 and 1.29 × 10^−3^, respectively, at a frequency of 5 GHz.

**Figure 1:**
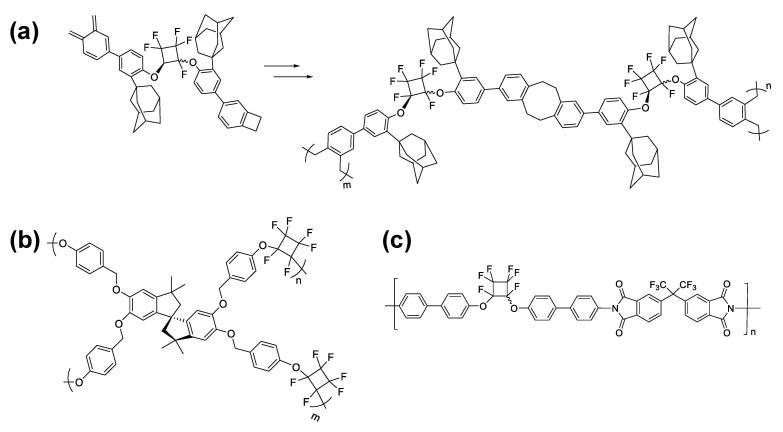
Structures of (**a**) crosslinked PFCB polymer from a monomer obtained by combining adamantyl, PFCB, and benzocyclobutene groups [31], (**b**) PFCB-containing spiro-centered polymer [32], and (**c**) PFCB-based polyimide [33].

Kong et al. [31] also synthesized a crosslinked PFCB polymer (structure shown in Figure 1a) from a monomer obtained by combining rigid adamantyl, PFCB, and benzocyclobutene groups. The introduction of PFCB can modify the polarizability and surface energy of the polymer, while the presence of rigid adamantyl groups can affect the free volume and density of the resulting material. The obtained polymer showed high thermal stability, hydrophobicity, low D_k_ (2.38), and low dielectric loss (<0.001) in a wide range of frequencies. Wang and coworkers [32] synthesized a spiro-centered perfluorinated polymer (Figure 1b) containing PFCB arms via a conventional [2 + 2] cyclodimerization above 150 °C. Spiro-centered polymers, owing to the presence of spiro-moieties across the chain, are robust, high-performance materials with excellent thermal stability, mechanical properties, and good processability [32,69]. The resulting polymer showed high thermal resistance, low D_k_, and low water uptake. Furthermore, Jia et al. [33] synthesized a BP-PFCB aryl ether-based polyimide (Figure 1c) from PFCB-based diamine and fluorine-containing dianhydride monomers via a two-step process. Results showed that the polymeric material exhibited outstanding moisture resistance and low D_k_ value, which are attributed to the polymer’s hydrophobicity and small free volume fraction, respectively.

### 2.3. PFCB-Based Polysiloxanes

**Scheme 5:**
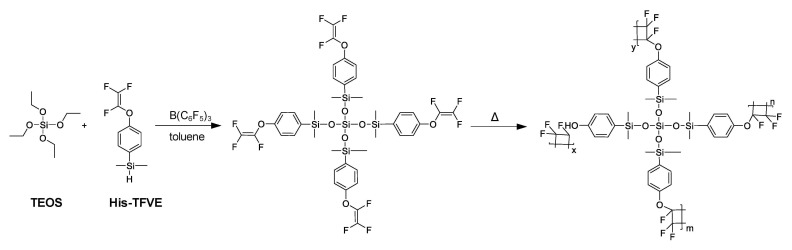
Synthesis of PFCB-containing polysiloxane [68].

Fluorinated polysiloxanes are an important class of high-performance materials with good processability and high thermal stability [28,36,68]. They are generally prepared via the (1) ring opening of fluorinated siloxane monomer and (2) hydrosilylation reactions between polysiloxane monomers and fluorinated compounds [28,68]. Wang et al. [68] reported a novel fluorinated polysiloxane macromonomer synthesized from tetraethoxysilane (TEOS) and TFVE-containing arylhydrosilane (HSi-TFVE) via a one-step Piers Rubinsztajn reaction using B(C_6_F_5_)_3_ (Scheme 5). The synthesized fluorinated TEOS monomer was then polymerized to form a cross-linked PFCB-containing polysiloxane network.

**Scheme 6:**
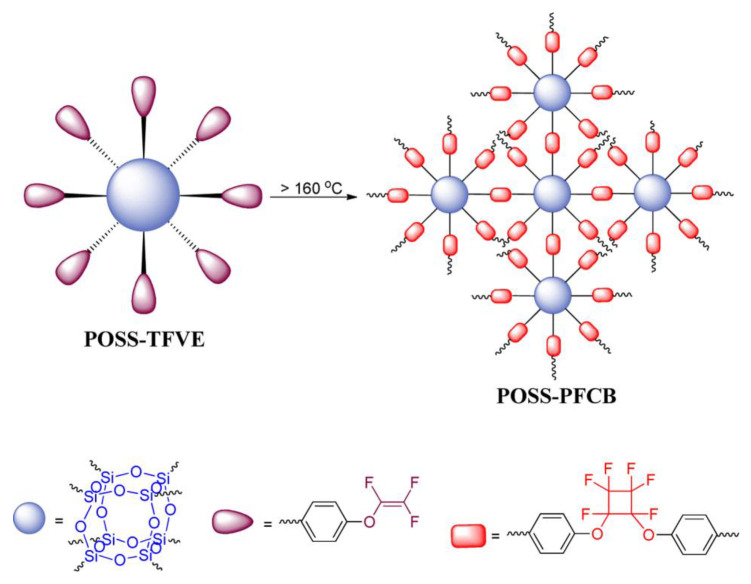
Preparation of POSS-PFCB via heat-induced cycloaddition of POSS-TFVE. Reprinted with permission from ref. [66]. Copyright 2017 ACS.

PFCB-based POSS have also become popular because they exhibit low surface energy, hydrophobicity, thermal stability, mechanical toughness, and chemical resistance [65,66]. POSS molecules are made of a silicon-oxygen core and feature cage-like structure of organic groups. Well-known for their excellent thermal and physico-chemical properties, POSS systems can be easily introduced by physical mixing and/or chemical bonding into the polymer matrix to form nanostructured hybrid organic−inorganic materials [65,66]. Wang et al. [66] synthesized a fluorinated functional POSS monomer containing thermally crosslinkable TFVE groups via a Pt-catalyzed hydrosilylation between octavinylsilsesquioxane and TFVE-containing arylhydrosilane (Scheme 6). The resulting organic-inorganic hybrid PFCB-containing POSS obtained by thermal [2 + 2] cycloaddition reaction showed high thermal stability, good film transparency, and low water uptake.

### 2.4. PFCB-Based Polymers from Renewable and Biobased Materials

**Scheme 7:**
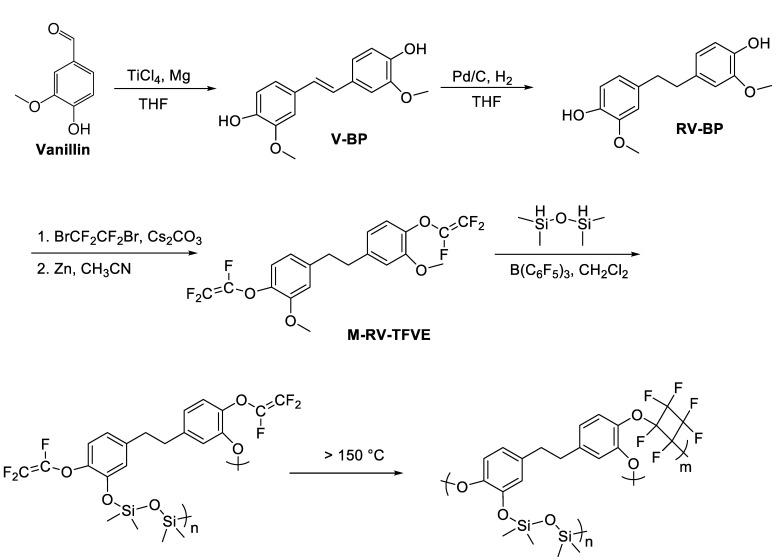
Synthesis of biobased PFCB-containing polysiloxane from vanillin [60].

In pursuit of sustainability, Tao et al. [60] synthesized a high-performance PFCB-containing polysiloxane using vanillin as starting material. As shown in Scheme 7, the aldehyde groups of vanillin were first transformed into unsaturated bisphenol (V-BP) through a McMurry coupling reaction, followed by a double bond reduction in a Pd/C catalyst to obtain saturated bisphenol (RV-BP), and introduction of TFVE groups. The resulting monomer (M-RV-TFVE) was reacted with disiloxane in B(C_6_F_5_)_3_ and subsequently cured above 150 °C to obtain the PFCB-based polysiloxane thermoset. It is noteworthy that the presence of PFCB groups provided the crosslinkable polysiloxane polymer with good thermal stability and chemical resistance.

**Scheme 8:**
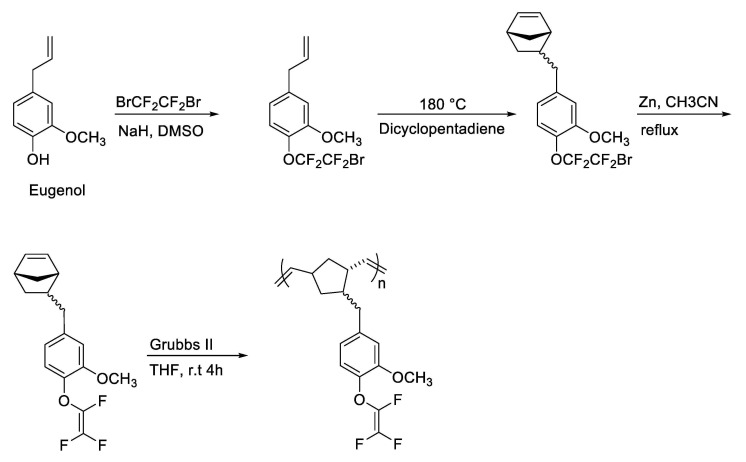
Synthesis of norbornene, TFVE-containing monomer from eugenol [61].

Fang et al. [61] recently reported the utility of eugenol to produce a new norbornene, TFVE-containing monomer, which can be polymerized into a crosslinked network containing PFCB moieties (Scheme 8). Eugenol has become popular as a renewable bio-feedstock for the synthesis of new high-performance materials due to its ready availability at low cost and reactive hydroxyl and unsaturated functional groups [70]. Note that the hydroxyl can be reacted to introduce fluorine-containing groups, while the double bond can undergo a Diels-Alder reaction with dicyclopentadiene. The resulting biobased PFCB polymer displayed good thermal stability, film transparency, and dielectric property.

### 2.5. Amphiphilic PFCB Polymers

**Scheme 9:**
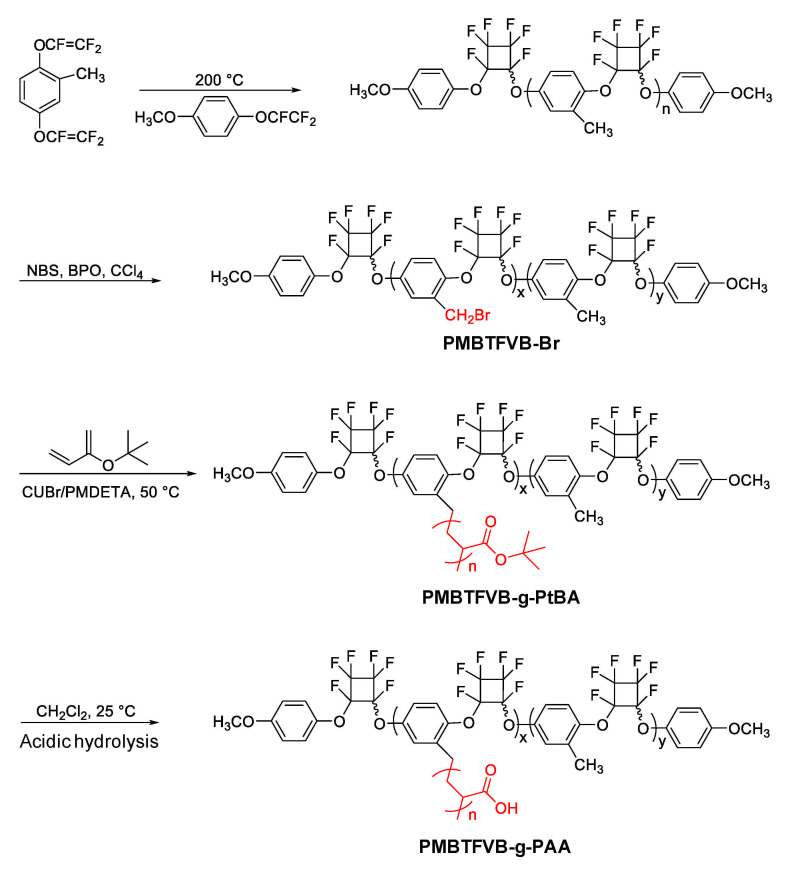
Synthesis of amphiphilic graft copolymer with PMBTFVB backbone and PAA side chains [62].

Amphiphilic copolymers are another class of macromolecules, which exhibit microscopic phase separation and self-assembly behavior at interfaces and in solution, and are mostly used as solubilizers and in drug delivery, catalysis, and packaging materials for electronics [63,71]. Among the amphiphilic copolymers, the fluorine-containing ones are popular because tailored physicochemical properties can be obtained for high-performance applications [62,63]. Liu et al. [62] prepared amphiphilic, brush-type PFCB-based graft copolymers composed of a semi-fluorinated poly(2-methyl-1,4-bistrifluorovinyloxybenzene) (PMBTFVB) backbone and hydrophilic poly(acrylic acid) (PAA) side chains through the combination of thermal cycloaddition polymerization and atom transfer radical polymerization (ATRP). As shown in Scheme 9, the target PMBTFVB-g-PAA was prepared by acidolysis of poly(tert-butyl acrylate) (PtBA) side chains of PMBTFVB-g-PtBA, which was obtained via ATRP of tBA using PMBTFVB-Br as macroinitiator. Results showed that the synthesized polymers displayed narrow molecular weight distributions and can self-assemble into peculiar nanostructures in aqueous media due to their structural features, which combine the flexible and robust aromatic ethers with fluorocarbon linkages.

**Scheme 10:**
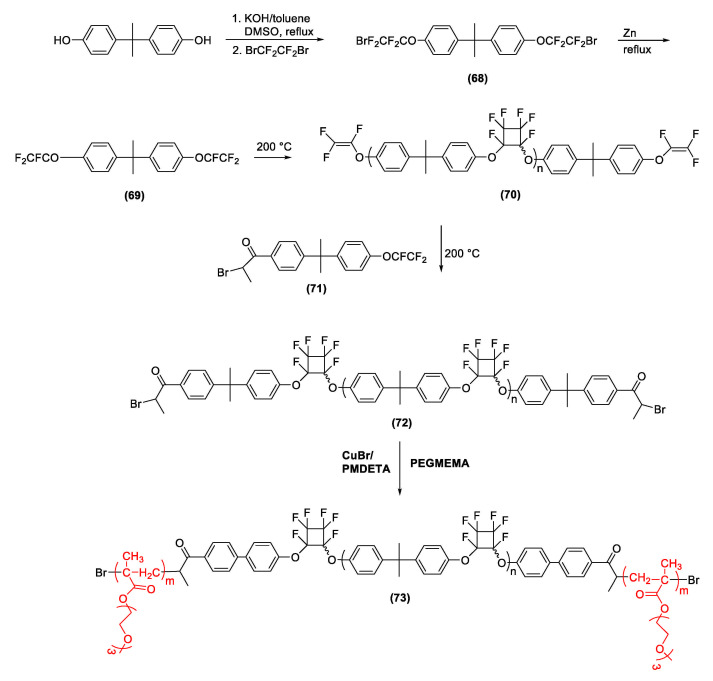
Synthesis of amphiphilic block copolymer with PFCB center block and PEGMEMA side blocks [63].

Feng et al. [63] also synthesized an amphiphilic block copolymer containing a hydrophobic PFCB center block and hydrophilic poly(ethylene glycol) methyl ether methacrylate (PEGMEMA) side blocks via thermal step-growth cyclopolymerization, followed by ATRP (Scheme 10). It can be seen that the polymer product showed two different structural segments with distinct solubility and self-assembly behavior in water. This specific amphiphilicity provided the block copolymer with the ability to form nanostructures in aqueous media via self-assembly.

### 2.6. Sulfonated PFCB-Based Polymers

**Scheme 11:**
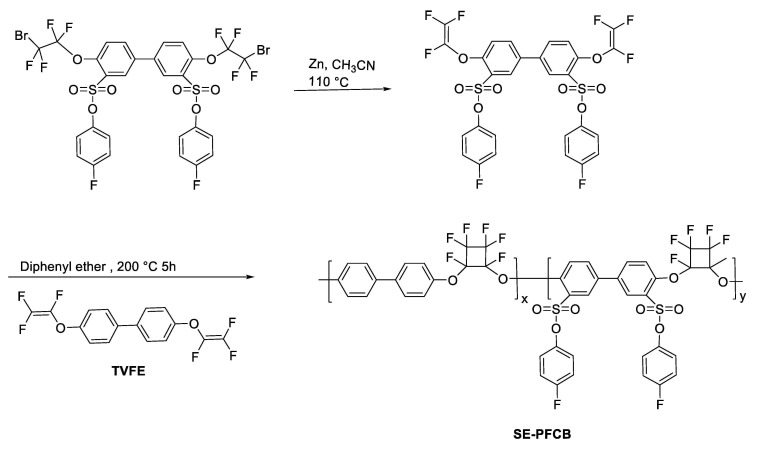
Synthesis of sulfonate ester-containing PFCB [27].

The incorporation of proton conducting groups such as sulfonimide and sulfonic acids in PFCB backbones via direct sulfonation has recently been investigated for the fabrication of fuel cell membranes [25,26,27]. Direct sulfonation is an effective method because it allows smooth inclusion of sulfonic acid groups, leading to a wide variety of sulfonated PFCB polymer structures. Marestin et al. [27] synthesized a sulfonate ester-containing PFCB (SE-PFCB) polymer by direct polycondensation of a bis(trifluorovinyl ether) precursor containing sulfonic acid groups protected as sulfonate esters with 4,4′-bis(trifluorovinyloxy) biphenyl (Scheme 11). The obtained soluble sulfonic-acid-containing PFCB polymer showed promising proton conductivity. Qian et al. [72] also synthesized PFCB-containing polybenzimidazoles from PFCB diacid and tetraaminobiphenyl in a solvent mixture composed of methanesulfonic acid and phosphorus pentoxide. Combined with high molecular weight and high thermal stability, the resulting PFCB polymer can be used to fabricate membrane electrode assemblies for fuel cell applications.

**Scheme 12:**
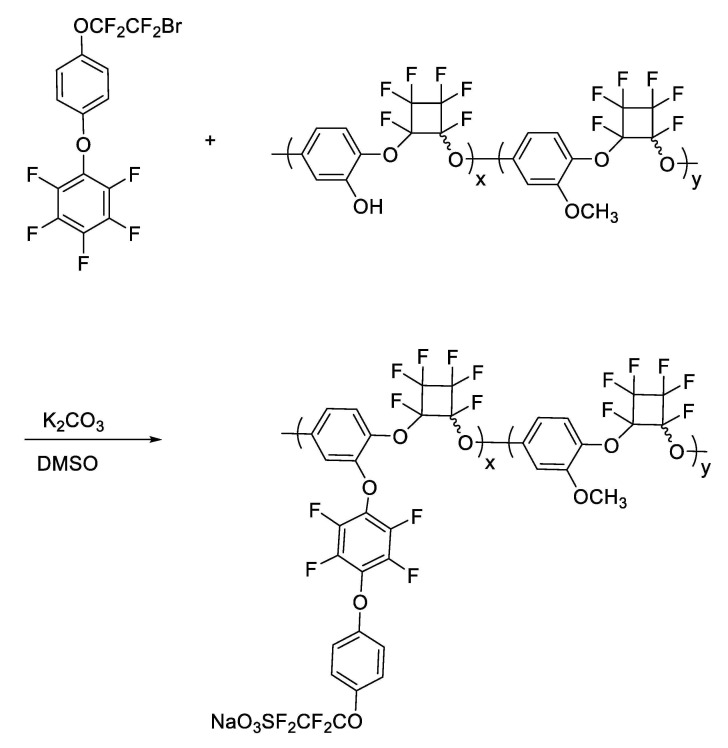
Synthesis of pendant perfluoroalkyl sulfonated PFCB polymer [73].

Acid-functionalized aromatic hydrocarbon polymers containing superacids can also be used as polymer electrolytes due to their enhanced conductive properties [73,74]. Bi et al. [73] incorporated a superacid group into a PFCB polymer by chemical post-modification of the PFCB polymer containing hydroxyl moieties with a perfluoroalkyl sulfonated side-chain precursor through a nucleophilic aromatic substitution reaction (Scheme 12). The resulting pendant perfluoroalkyl sulfonated PFCB polymer exhibited high thermal stability and good solubility in polar solvents.

### 2.7. PFCBs with Polycyclic Aromatic Rings

**Scheme 13:**
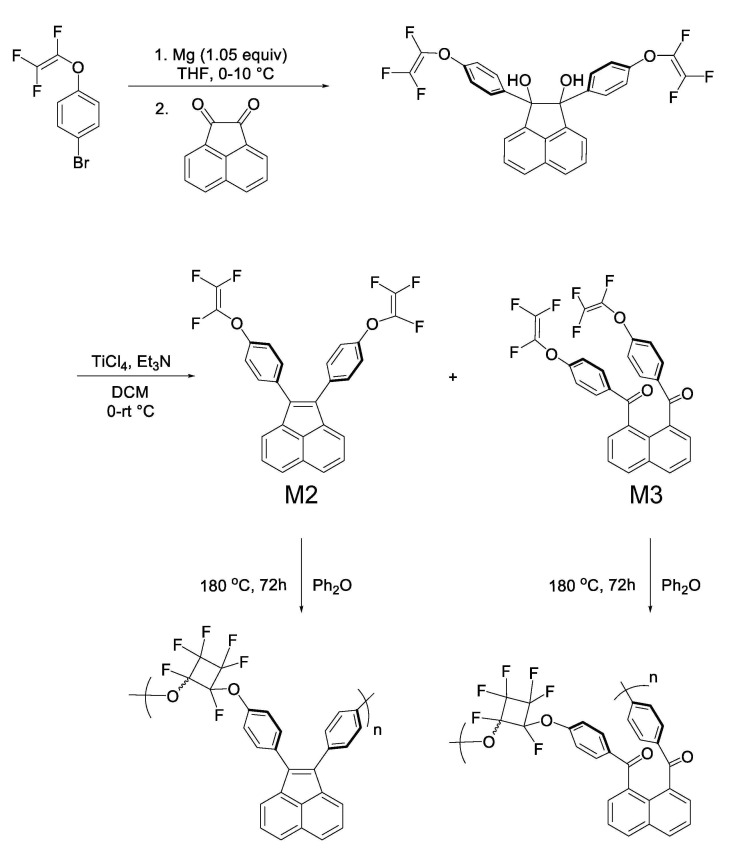
Synthesis of PFCB-based PAHs from acenaphthylene derivatives [48].

**Figure 2:**
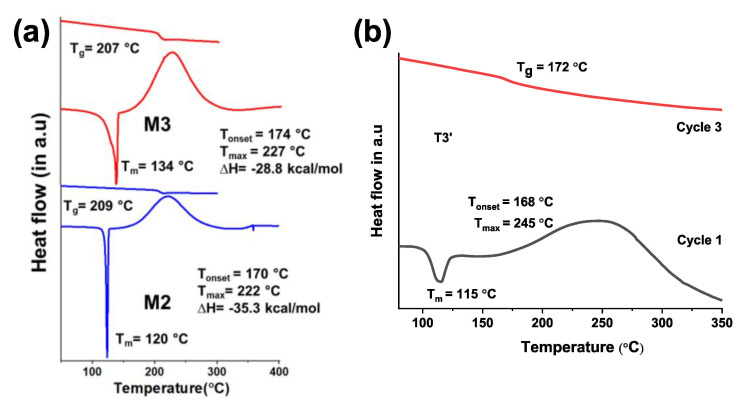
Differential scanning calorimetry (DSC) analyses for (**a**) M2 and M3 in Scheme 13 (Reprinted from [48] with permission from Wiley) and (**b**) T3′ in Scheme 14 (Reprinted from [49] with permission from Elsevier).

**Scheme 14:**
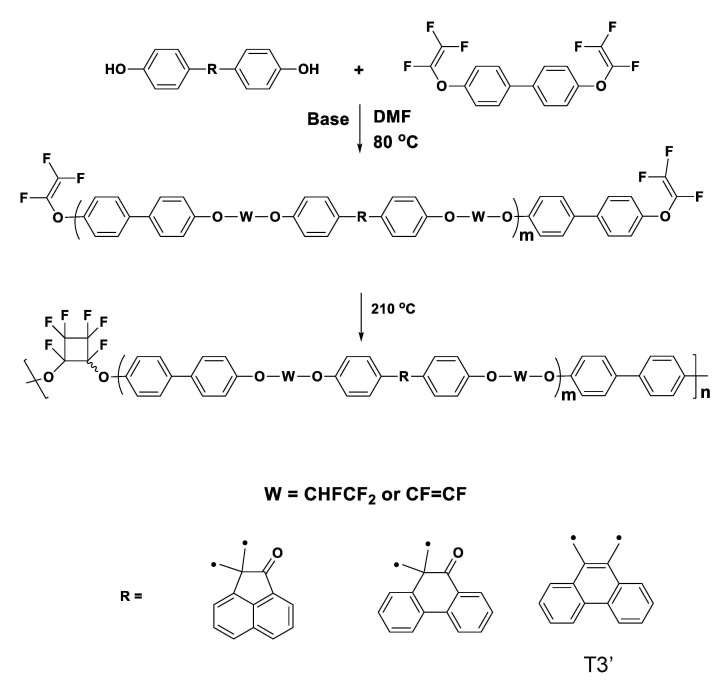
Preparation of TFVE-end capped PAHs and their thermal chain extension to PFCB polymers [49].

Although PFCB polymers have been prepared from TFVE aromatic monomers containing a wide range of different functional groups, only a few studies have reported PFCBs with extended or fused aromatic segments [47,64]. Recently, our group has made efforts in synthesizing PFCBs containing large polycyclic aromatic hydrocarbons (PAHs) [48,49]. Displayed in Scheme 13 are the PAH-containing PFCB polymers prepared from TFVE precursor monomers using inexpensive acenaphthylene derivatives [48]. The resulting PFCB polymers can be easily formed into thin films with high molecular weight, glass transition temperature (*T_g_*) above 200 °C (Figure 2a), and thermal stability of up to 450 °C. Likewise, Scheme 14 shows the utility of bisphenol-based PAHs in the preparation of new PFCB polymers, which exhibited variable *T_g_* values and high thermal resistance in both air and N_2_ [49]. As shown in Figure 2b, the first heating cycle of the telechelic polymer triggered two thermal events: a broad polymer endotherm occurring at 115 °C, followed by a slow polymerization onset at 168 °C, with a maximum at 245 °C. These PFCB polymers also contain intact enchained fluoroalkenylene moieties susceptible to further post-polymerization and crosslinking. In general, the inclusion of PAH cores into PFCBs results in polymers with higher thermal stability compared to polymers prepared from conventional precursors.

### 2.8. Applications

The versatility of the aryl ether linkages, presence of thermally stable aromatic rings, and improved processability due to the PFCB ring make the utility of PFCB polymers promising in many practical applications [9,11,20,21,22,23,24,25,26,27,28,29,30,31,32,33,34,35,36,37,38,39,40,41,42,43]. This section summarizes several important uses of PFCBs, such as high-performance protective coatings, membrane layers, dielectrics, and optical materials.

#### 2.8.1. Surface Coatings

High molecular weight PFCB polymers can be easily melt- or solution-processed [11]. The resulting melt or polymer solution can be either spin or dip coated and cured above 150 °C to give thermally stable, nonstick, and optically clear protective films [11,20,21,22,65]. Caldona et al. [20] recently showed, for the first time, the utility of three commonly used PFCBs, one crosslinked (TP) and two linear (BP and 6F), as corrosion preventing coatings for mild steel. The chemical structures of these PFCBs are shown in Scheme 1a. Results via electrochemical measurements and accelerated corrosion tests in chloride-containing solutions revealed that the PFCB polymers displayed corrosion protection for mild steel with impedance values of up to 10^6^ Ω cm^2^, nearly equivalent to that of a commercial polyvinylidene fluoride (PVDF) coating. The PFCB polymer coatings also displayed high thermal stability, nonstick property, hydrophobicity, and good adhesion to metal surfaces.

Zhou et al. [21] optimized the preparation of BP PFCB polymer films by investigating the effects of solvents, polymer solution concentration, and dip coating speed on the resulting film uniformity and thickness. Analyses showed that the: (1) films formed were generally uniform, smooth, and defect-free; (2) film thickness was dependent on both polymer concentration and withdraw speed; (3) water contact angles (CA) did not vary with film thickness; and (4) films formed using THF and chloroform solvents were uniform, while those formed using acetone were not. These results provided guidelines to the authors for their subsequent studies on the fabrication of PFCB membrane materials for gas separations, which are further reviewed in Section 2.8.2.

Due to their hydrophobic character, PFCBs and their composites also find useful applications in the preparation of controllable liquid-repellent surfaces and materials. Iacono et al. [65] prepared fluoropolymer composite films composed of a 6F PFCB polymer as the matrix and varying loads of fluorinated POSS (F-POSS) as the modifying agent. The resulting pristine 6F PFCB polymer film was hydrophobic with a water CA of ~95°, but demonstrated oleophilicity with a hexadecane CA of ~31°. The inclusion of F-POSS as surface modifier increased both water and hexadecane repellency, giving maximum CA values of 124° and 80°, respectively, at F-POSS loadings of 10–15 wt%. This anti-wettability enhancement can be credited to the synergistic effect of the low surface energy of both the PFCB and fluorinated silsesquioxanes and the surface roughness caused by F-POSS dispersion over the PFCB polymer matrix. The preparation of PFCB-based POSS and polysiloxanes has been discussed in Section 2.3.

Verma et al. [22], on the other hand, fabricated a BP PFCB polymer surface doped with less than 1 wt% of 1-butyl-3-methylimidazolium hexafluorophosphate, a room temperature ionic liquid, by electrospinning process. Compared to a CA of ~90° for the undoped form, the electrospun ionic-liquid-doped PFCB exhibited a superhydrophobic behavior with a CA of 154 ± 2°. The authors attributed this water repellency increase to the presence of the ionic liquid, which (1) introduced the roughness by creating nanostructures on the PFCB surface and (2) further lowered the surface energy by increasing the fluorine content of the PFCB matrix. Overall findings suggest that the inclusion of such additives or dopants does not compromise the bulk properties of the PFCB polymer matrix [22,65], thus, making PFCBs attractive for applications in liquid-repellent composites for coatings and fibers.

#### 2.8.2. Membrane Layer Materials

PFCB polymers are also good candidate materials for the development of composite membranes for gas separation and fuel cell applications due to their rich-fluorine backbone and architectural structure capable of introducing free volume to the polymer [23,24,25,26,27]. In general, PFCBs possess amorphous structure and exhibit remarkable processability, both of which promote the ease of fabricating membrane composite materials.

One of the earliest studies related to the utility of a PFCB polymer in fabricating gas membrane materials were conducted by Zhou and coworkers [23,24]. The performance of a BP PFCB polymer as selective membrane layer for CO_2_ gas separations was first described [23], in which the fabricated support membranes with varying thickness of PFCB polymer films gave CO_2_ permeance values of 200–1700 GPU and a CO_2_/N_2_ selectivity value as high as 20. Although these results suggest the potential use of PFCBs in membrane gas separations, the moisture content of gas streams, particularly at saturation levels may reduce the membrane gas permeance, leading to a decreased performance over time.

In order to account for the stability, long-term effect, and robustness of the separation membranes, the CO_2_ plasticization and physical aging of the PFCB selective layers were further examined [24]. Plasticization is caused by membrane swelling as a result of gas absorption by the polymer, leading to a reduced selectivity. It was observed that more concentrated PFCB polymer solutions for film thicknesses >40 nm were found to display higher plasticization resistance, but with reduced permeance at higher annealing temperatures. In addition, continued use of a plasticized polymer layer encourages physical aging on the membrane material, which may affect the free volume and permeability. Overall, these results are thought to be useful in guiding further developments on the use of PFCB polymers as components for gas separation membrane materials.

The thermal-oxidative durability of PFCBs also affords their potential use as polymeric materials for fuel cell membranes [25,26,27]. Kalaw et al. [25] prepared blends of hydrophobic and sulfonated hydrophilic BP PFCB polymers to combine both the thermally and mechanically stable backbone of PFCB polymer and the acidic sulfonated PFCB, thereby mimicking the phase-separated structural features of Nafion^TM^. A 1:1 mole ratio polymer blend gave an ion exchange capacity (IEC) of ~1.37 mmol/g, roughly 1.5 times higher than that of Nafion^TM^, and a proton conductivity of up to 0.15 S cm^−1^ at 100 °C. Chang et al. [26] also fabricated a membrane of poly(fluorene-co-sulfone)ethers containing PFCB groups, which exhibited nearly equivalent IEC and proton conductivity values of 1.83 meq/g and 3.5 × 10^−2^ S cm^−1^, respectively.

#### 2.8.3. Electrical Applications

The presence of partially fluorinated blocks yields polymers characterized, not only by chemical, moisture, and thermal resistance, but also by low D_k_. Hence, PFCB-containing materials also find applications in electrical insulations [28,29,30,31,32,33], polymer light-emitting diodes (PLEDs) [34,35,36], and electrochromic devices [37].

**Figure 3:**
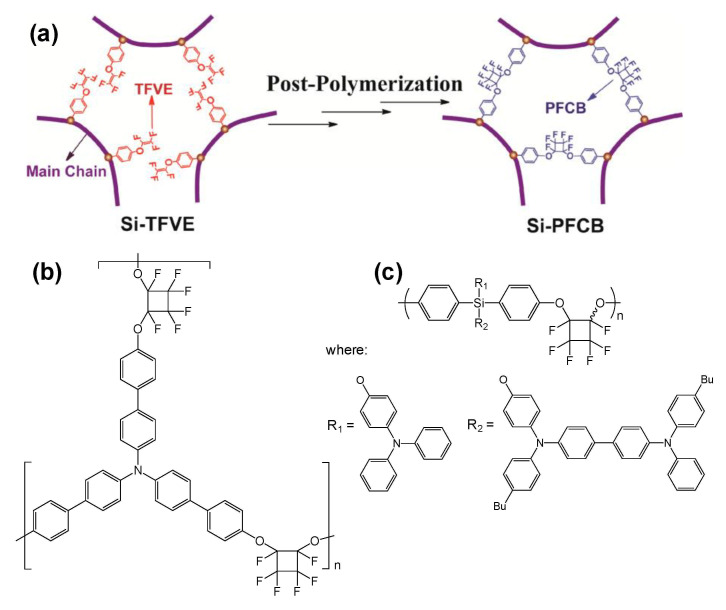
(**a**) Preparation of amorphous polysiloxane crosslinked with PFCBs. Reprinted from [28] with permission from ACS. Structures of (**b**) PFCB-containing crosslinked polyarylamine [34] and (**c**) BTPD-Si-PFCB as functional HTMs [35].

Yuan et al. [28] synthesized an amorphous polymer with a siloxane backbone crosslinked with PFCB groups (Figure 3a). The measured D_k_ for the resultant PFCB-containing polymer at frequencies of up to 30 MHz was 2.33, which is lower than those of commonly used low-D_k_ materials such as polycyanate esters, polyimides, and other Si-containing resins. Ghim et al. [29] also reported the synthesis and utility of a thermosetting PFCB-based polymeric material as gate dielectric for an organic thin film transistor, which displayed promising electrical properties in mobility and on/off current ratio.

The use of PFCBs as hole-transporting materials (HTM) in PLEDs has also been reported in the literature [34,35,36]. Lim, Heeger (who won the 2000 Nobel Prize in Chemistry for his work on conductive polymers), and co-workers [34] synthesized a crosslinkable arylamine containing PFCB groups (Figure 3b) and characterized its capacity to act as a hole-transporting layer. The resulting PFCB-containing HTM was highly transparent, solvent resistant, thermally stable, and displayed a larger turn on voltage than that of a poly(3,4-ethylenedioxythiophene) polystyrene sulfonate (PEDOT-PSS) based material. In another study, Jiang et al. [35] developed a series of HTMs based on PFCBs containing triarylamine side chains. These PFCBs are characterized by the energy of the highest occupied molecular orbital (*E_HOMO_*) that matches the work function for indium tin oxide (ITO), an anode commonly employed for typical LEDs. Among the series of PFCB-based materials examined, bis(N,N′-diphenyl-N,N′-bis(3-butylphenyl)-(1,1′biphenyl)-4,4′-amine-PFCB (BTPD-Si-PFCB) (structure shown in Figure 3c) exhibited the most promising hole-transporting activity such that highly efficient green-, red-, and blue-emitting PLEDs have been developed. Likewise, Gong et al. [36] showed the use of BTPD-Si-PFCB as HTM for PLEDs and its performance has been observed to be nearly equivalent to that of the PEDOT-PSS.

Lim et al. [37] investigated the application of PFCB polymers in electrochromic devices by synthesizing N,N,N′,N′-tetraphenyl-biphenyl-4,4′-diamine (TPD) containing a PFCB group and characterizing the polymer’s electrochromic properties. Based on the spectroelectrochemical results, the oxidation of initially colorless TPD-PFCB films resulted in a color change to yellow and subsequently to greenish blue, while showing an electrochromic coloration efficiency of up to 602 cm^2^ C^−1^. In general, the presence of PFCBs in organic electrochromic chromophores, not only improves their chemical stability, but also increases their optical transparency and thermal stability [37].

#### 2.8.4. Optical Applications

In addition to their wide array of beneficial and tailorable properties, PFCBs are among the few known polymeric materials that exhibit low optical attenuations in the wavelength range of 1300–1550 nm [11,38,39]. Thus, PFCBs have become attractive candidate materials for a variety of optical and photonic devices.

Ballato and co-workers [40] have reported and established the optical properties of BP and 6F PFCB polymers by measuring the refractive index (RI) and extinction coefficients over a wide spectral range of 0.13–33 μm. Results showed that the PFCB polymers exhibited RI of >2.0 near a wavelength of 10 μm and low extinction coefficients in the atmospheric transmission, mid-infrared, and a portion of ultraviolet regions. Ghim et al. [41] also studied the optical properties of naphthalene-based PFCB in the form of films and optical fibers. The presence of naphthalene introduces a rigid structure, resulting in a relatively high glass transition temperature (*T_g_*) polymer. The attenuation loss and birefringence values were as low as 0.083 dB cm^−1^ at 910 nm and 0.0005, respectively. In comparison, PFCB copolymers, which can exhibit highly improved and tailored properties, have been found to display RI values in the range of 1.4–1.5 at 1550 nm wavelength and *T_g_* of 350 °C, and RI birefringence as small as 0.003 [9]. The high *T_g_* and low birefringence values are desirable characteristics for integrated optic applications as they can alleviate thermomechanical distortions and eliminate any restrictions on dispersion-related bandwidth. An inexpensive micro-transfer molding technique was also employed for the PFCB copolymers and their use as polymer waveguides and optical clad layers in microphotonics has been demonstrated [42]. All these reports suggest PFCBs as promising materials for the fabrication of photonic devices.

Actual photonic devices composed of PFCBs were physically fabricated to illustrate the fluoropolymer’s practical functionality in such applications. Planar photonic structures capable of functioning in the visible and near-infrared spectral regions were prepared by Shah et al. [38] by patterning microscopic diffractive linear gratings into a BP PFCB polymer film through direct micromolding using a silicon master. Diffraction effects, particularly from the green to red spectral portions of the visible region, were observed from the photonic structure, with a bandwidth of 3 dB at roughly 30 nm.

Bragg reflective wavelength filters, operating at a wavelength of 1.55 μm, were constructed by Oh et al. [43] using PFCB polymer as a cladding layer. Results showed a reflectivity of 30 dB at the Bragg wavelength, a 3.7 dB insertion loss, and a 0.6-nm 3 dB bandwidth. These reflective filters can be combined with other waveguide devices to exploit wavelength division multiplexing and other useful optical applications. Arrayed-waveguide grating (AWG) structure, a moderately complex photonic device, was also fabricated by Jiang et al. [39] using PFCBs as both waveguide and cladding layer materials. The photonic device exhibited a 6–9 dB insertion loss for both fiber-to-chip and on-chip coupling, roughly −20 dB crosstalk, and a ±0.5 dB uniformity over the channels. In general, this PFCB-based photonic device offers simple solutions for an array of optical applications as it also exhibits lower thermal sensitivity compared to other polymeric materials and a central wavelength shift of less than 7 × 10^−2^ nm °C^−1^.

## 3. ODA-Derived Thermosets

**Scheme 15:**
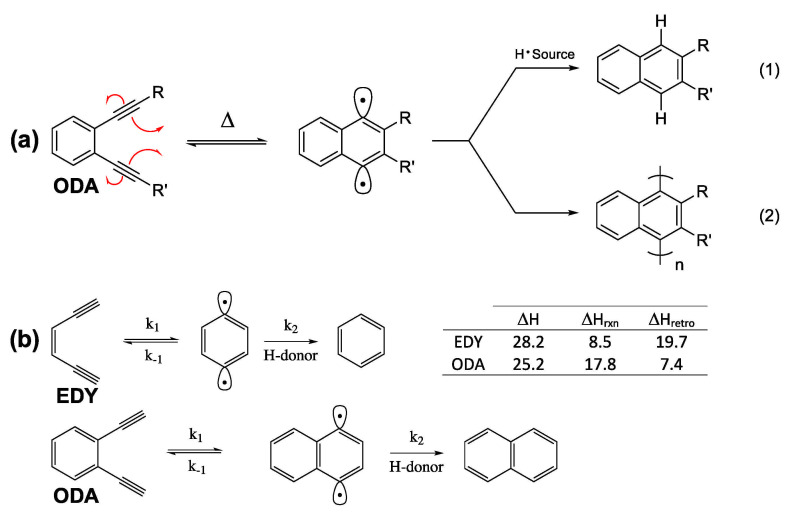
(**a**) Thermal cycloaromatization of ODA via the Bergman reaction in the (1) presence of abstractable heteroatoms/proton donors or (2) absence of such species, being either in solution or melt [75]. (**b**) EDY and ODA monomers with their corresponding experimental Bergman cyclization (ΔH_rxn_), retro-cyclization (ΔH_retro_), and proton abstraction energy barriers (ΔH) in kcal mol^−1^ [76].

*Ortho*-diynylarenes (ODAs) are aromatic enediynes (EDY), which can undergo a unique diradical-mediated, thermal step-growth polymerization (i.e. Bergman cyclization) to yield thermally stable, conjugated organic polymers (structure (2) in Scheme 15a) [75]. The difference between the traditional EDY and ODA monomers lies in the thermodynamics of their cyclization (Scheme 15b). The ODA monomer, being the benzannulated analogue, has a lower barrier for retro-cyclization compared to that of the linear EDY [76].

Like PFCBs, ODA-derived polymers are synthesized via a thermally initiated, ring-closing polymerization that offers several benefits: the chemistry (1) proceeds without the presence of exogenous chemical catalysts or reagents, (2) results in a high atom economy as the reaction undergoes a non-condensation process without forming volatile by-products, (3) produces polymers amenable to film-forming vapor deposition processes, and (4) includes a versatile substrate scope as a variety of monomers can be rapidly synthesized by well-established, facile metal-catalyzed coupling reactions. However, unlike the radical-mediated [2 + 2] cycloaddition of TFVEs, the polymerization of ODA monomers proceeds via a Bergman cycloaromatization to yield highly reactive aryl diradicals capable of (1) abstracting protons, (2) in the absence of a proton source, initiating other cyclization or propagation events (Scheme 15a) [76]. Although the Bergman cyclization reaction is extensively studied in the literature, within the scope of polymer science, it has been largely underutilized until recent years [77]. While the utility of Bergman cyclization in achieving novel, functional polymers has been reviewed in the literature [76,77], this section focuses on the synthesis, properties, and applications of ODA-derived thermosets and carbon.

### 3.1. Synthesis of ODA Monomers

In the mid-1980s, naturally occurring EDY-containing antibiotics, such as calichemicins [78] and esperamicins [79], were studied for antitumor activity. Their ability to abstract hydrogens from a DNA backbone, resulting in DNA cleavage or crosslinking events that trigger cell death, merited further investigation on EDYs as an emerging class of powerful cytotoxic motif [80]. The renaissance of studies in this area prompted researchers to synthesize a variety of non-natural EDY analogues, thereby establishing a reliable synthetic route for this unique class of monomers.

**Scheme 16:**
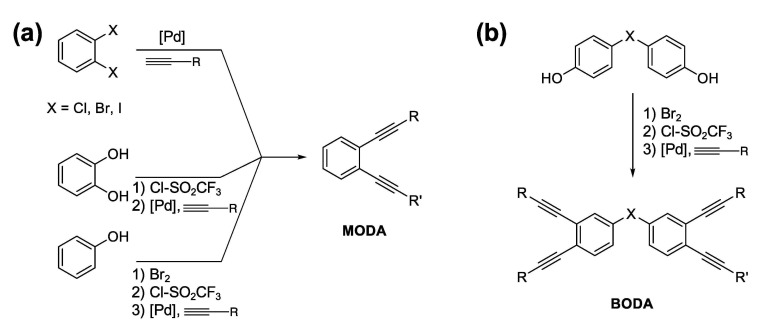
General synthesis route for (**a**) MODA and (**b**) BODA monomers using commercially available precursors. [Pd] = Sonogashira conditions. Cl-SO_2_CF_3_ = Triflate esterification.

The Sonogashira coupling is one of the most reliable methods of producing distinct acetylenes [81,82,83]. It involves the coupling of aryl or vinyl halides with terminal alkynes using Pd (II) and cuprous iodide. Therefore, its utility has provided many artificially based EDYs for medicinal use [84,85] and has become the predominant synthetic route for ODA monomers (Scheme 16). Since its inception, the Sonogashira methodology has been developed to proceed via microwave, which significantly reduces a 24-h reaction time to only 3 min [86]. Activated Pd, obtained from a mixture of Pd(PPh_3_)_2_Cl_2_, and an excess of magnesium in pyridine, was shown to be required in less equivalents (i.e., 0.001–0.0001) [87]. Other advances in the reaction conditions and scope of Sonogashira substrates have been widely reviewed in the literature [83].

Mono- and bis-*ortho*-diynylarenes (MODA and BODA) are the nomenclatures assigned to the bifunctional and crosslinkable monomers, respectively. MODAs can be easily synthesized from *ortho*-dihaloaromatic precursors (Scheme 16a) in a one-step Sonogashira coupling. Typically, bromo- and iodoaryl substrates are preferred, but a modified Sonogashira reaction can also make use of chlorinated aryl substrates [88] for access to a larger pool of starting materials (e.g., polychlorinated biphenyls). Likewise, naturally based catechols are inexpensive precursors, requiring a two-step conversion to MODA (Scheme 16a) [89].

While MODAs can be utilized for several applications [51,77,90], within the context of thermosetting BODA-based polymers, they serve as reactive diluents to control branching, solubility, and processability. Although BODA homopolymers require relatively high molecular weight for favorable viscosity in solution or bulk in some applications, polymerization to higher molecular weights risks crosslinking. Copolymerization with a bifunctional MODA can change the degree of crosslinking, modify the gelation time, and viscosity, and result in an equivalent structural backbone that yields all of the same desirable properties (e.g., carbon yield).

BODAs, on the other hand, are tetrafunctional monomers used for producing branched oligomers with variable molecular weight, polydispersity, rheological properties, and large processing windows towards crosslinked networks. Prior to curing, BODAs display excellent solution and melt processability as their melting points typically lie sufficiently below the onset of polymerization [52,87]. Precursors like those used in the synthesis of MODAs can be used to prepare BODA monomers as well. For example, Jones and Keller [87] synthesized 1,2,4,5-phenylethynylbenzene and a series of other thermally cross-linkable *meta*- and *ortho*-phenylethynylbenzenes by the Pd-catalyzed coupling reaction of tri- and tetrabromobenzenes with phenylacetylene. Alternatively, BODA monomers have been prepared in large scales with good yields by selective *o*-bromination of inexpensive, commercially available bisphenols, followed by triflation and Sonogashira coupling (Scheme 16b) with terminal alkynes [52].

Moreover, the structural versatility of the crosslinkers offers exceptional control in properties. For instance, variable X spacers can alter the crystallinity or solubility [55], and impart thermal-oxidative stability [91]. Variable terminal R groups (Scheme 16b) can influence processability, conjugation, architecture, hydrodynamic volume, viscosity, and reactivity. This simplicity makes Bergman cyclization an attractive route to novel polymers, thermosets, and precursors to carbon structures.

### 3.2. Bergman Cyclopolymerization

**Figure 4:**
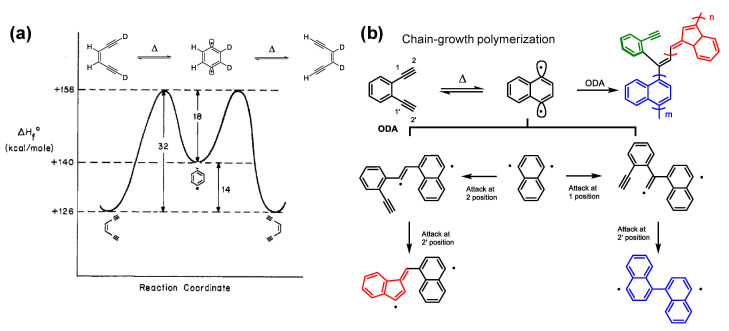
(**a**) Reversibility of EDY cyclization experimentally observed by the unimolecular deuterium scrambling and its reaction. Reprinted from [92] with permission from ACS. (**b**) Chain-growth polymerization of ODA monomers [57].

Bergman’s pioneering studies on EDYs revealed their unimolecular and reversible isomerization by observing the rapid deuterium scrambling of *cis*-3-hexen-1,5-diyne at 200 °C (Figure 4a). This study provided the first critical insight on the mechanistic nature of thermal-initiated EDY cycloaromatization [56,92,93]. The reactive didehydrobenzene intermediate, strongly suggested in these seminal studies [92], opened the possibilities to a radical-mediated polymerization.

Motivated by the seminal studies put forth by Bergman, John and Tour [94] carried out the first experiments using thermal Bergman cyclization for synthesizing linear polyphenylenes and polynaphthalenes. The in-situ generated diradical was allowed to combine as building blocks to obtain thermally stable conjugated polymers. Polydisperse, high molecular weight polymers were obtained using benzene as a solvent at 50–160 °C [95]. The final polymer yield after fractional precipitation was 50–90% with *M_n_* values of 1500–2500, polydispersity index (PDI) of 3-11, and excellent thermal stability (10% weight loss after 400–600 °C). Neenan and Whitesides [96] also synthesized various inexpensive polyethynylbenzenes with high thermal stability and unprecedented carbon yields. However, some of these compounds exhibited explosiveness upon purification by distillation, thereby limiting their applications. Therefore, the preparation of ODAs are more practical as comparable properties can be achieved.

Bergman cyclopolymerization can occur under thermal [94,95], photochemical [97], and catalytic conditions [98], obtaining crosslinkable, highly branched, and processable B-staged resins. The polymerization mechanism, however, is more complicated than that of the unimolecular cycloaromatization. Homopolymerization can occur both by a chain-[28] and step-growth mechanisms (Figure 4b) [57]. The concentration of monomer during polymerization greatly influences the cyclization route and addition of external radical initiators does not affect the polymerization efficiency.

Johnson et al. [57] synthesized polynaphthalene via the Kumada coupling and Bergman cyclization. The generated diradical intermediate, being low in concentration, reacts with the ODA monomers in the solution, leading to highly irregular 5- to 6-membered rings containing unreacted alkynes (Figure 4b). However, other studies on the solution and melt polymerization show that the change in molecular weight with time and monomer conversion follows that of the step-growth polymerization [94].

### 3.3. Applications

*Meta*-[96], *para*-[99], and *ortho*-alkynylarenes [94] have been used in the fabrication of organic-based electronics and high temperature materials for a myriad of engineering applications. Particularly, the *ortho*-alkynylarenes (e.g., ODAs) are interesting because their ring closure via the Bergman cycloaromatization provides a thermally controlled, efficient, and atom-economic method of accessing a higher order of aromaticity [92,97]. This aromaticity level increases the electron conjugation along the polymer backbone and thermal stability of the resulting polymer. The conversion of the initial triple bonds also makes these materials useful in generating highly carbonaceous materials with enhanced thermal and electronic properties.

Initially invented by the Dow Chemical Co. for dielectric applications and developed at Clemson University, high-carbon yielding, ODA-derived polynaphthalene networks have found potential applications in micro- and nanoelectromechanical systems (MEMs or NEMS) [54], the fabrication of solid and hollow carbon fibers [58], carbon-based photonic crystals [100], mesoporous carbon structures [101], and the like. In this section, the utility of BODA-derived polymers in electronics and micro-optics is reviewed.

#### 3.3.1. Electronic Applications

As electronic devices become smaller in size, the materials used in their fabrication must be carefully considered to optimize, not only the portability, but also their efficiency and reliability. Although decreasing the physical dimensions of current electronic components may allow transistors to operate with increased speeds, parasitic capacitance between circuit elements causes an overall signal delay and inefficient operation [102]. Interlayer dielectrics have been sought for mitigating such signal delays between interconnected circuit elements [103]. While a number of viable low-D_k_ materials exist, full breadth consideration of materials properties may reduce the options. Candidate materials for interlayer dielectric applications must simultaneously be amenable to industrial processing conditions, compatible with metal lines, and mechanically tough [50]. Furthermore, such materials should be characterized by low moisture uptake, *T_g_* well above the processing temperature, high thermal oxidative stability, and excellent solubility in a wide variety of solvents.

**Figure 5:**
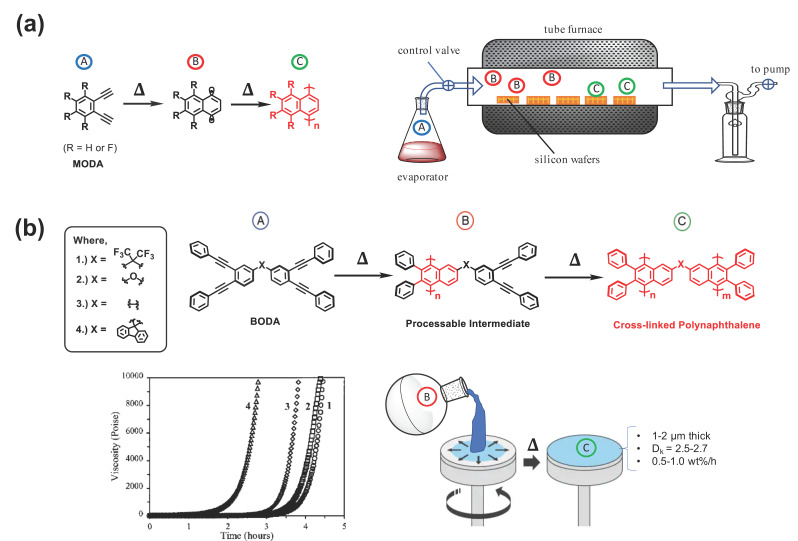
(**a**) Vapor deposition of high temperature, low-dielectric polynaphthalene thin films via gas phase thermolysis of ODA monomers [106]. (**b**) Schematic for the spin-coating of 1-4 derived B-staged resins (B) to a final cured thin film (C) with the reactive rheological data obtained isothermally at 210 °C. Reprinted from [52] with permission from ACS.

Organic polymeric materials naturally display low D_k_ values and can be synthetically tailored to meet the criteria for a specific electrical application [104,105]. However, matching both the performance and processability requirements is challenging. Although obtaining thermally stable thin films is important, inclusion of functional groups that impart thermal stability often leads to a decrease in the material’s solubility and processability. These issues can be overcome by using ODA-based materials. Due to their unique diradical chemistry, thin film formation is possible in linear, rigid, and sparingly soluble polynaphthalene polymers (Figure 5a) [106]. Specifically, polynaphthalene polymers can be vapor-deposited as thin films onto silicon wafers via gas phase thermolysis of ODA monomers [106,107,108,109,110].

Thermosetting BODA-derived polymers with improved processability were prepared by Smith et al. [52]. The step-growth polymerization of the tetraynes produced branched, solution-, and melt-processable oligomers with broad weight-average molecular weight (*M_w_*) of 25,000 and PDI of 11. As shown in Figure 5b, this processability provided ease in spin-coating thin films of controlled thickness (1–2 µm) with favorable D_k_ values of 2.5–2.7. Results further showed that the cured films exhibited thermomechanical stability of up to 450 °C, the highest processing temperature for typical microelectronics fabrication.

#### 3.3.2. Optical Applications

**Figure 6:**
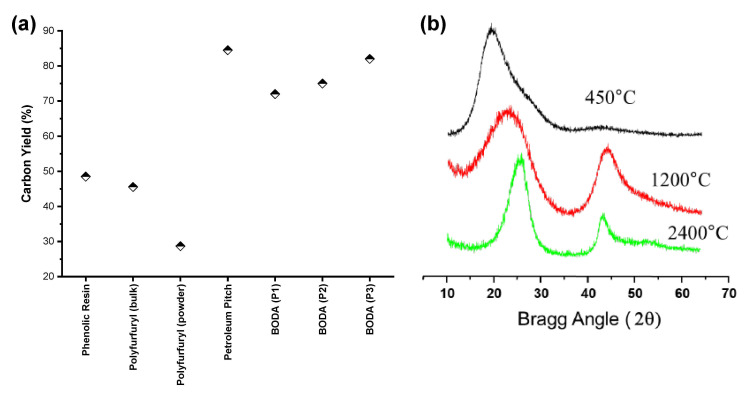
(**a**) Carbon yields obtained from [53,58,111,112,113]. (**b**) XRD spectrum of BODA taken at different temperatures. Reprinted from [111] with permission from Wiley.

The carbon yield of the highly aromatic thermosetting BODA-based polymers is relatively high compared to the typical polymeric precursors used for the manufacture of pyrolytic glass-like carbon (Figure 6a). Hence, such thermosetting polymers can be outstanding precursors for the fabrication of carbon microstructures and other carbon-related technology for optical applications.

**Figure 7:**
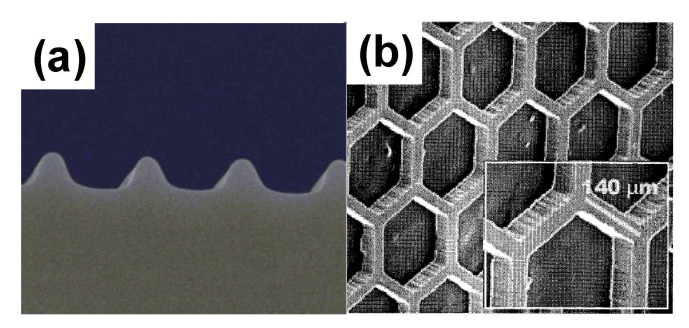
(**a**) BODA-derived glassy carbon submicrogratings. Reprinted from [111] with permission from Wiley. (**b**) Micromolded and patterned glassy carbon from BODA resin. Reprinted from [54] with permission from ACS.

Zengin et al. used BODA-derived carbons in fabricating submicrogratings for potential use in micro-optics and other optical microscale devices [111]. Resin 2 in Figure 5b was melt-polymerized to a moderately viscous intermediate at 210 °C for 3 h prior to being patterned on a silicon wafer-polydimethylsiloxane (PDMS) mold and cured at 300 °C. As shown in Figure 7a, the 0.4 µm spacing between the grating lines were isometrically reduced to a spacing of 0.3 µm and an overall uniform grating size of 0.5 µm was achieved after carbonization at 1000 °C under inert atmosphere, leading to a functional linear submicrograting. Note that the diffraction, reflection, and wavelength of light can be controlled both by the periodicity of the gratings and the light ray angle. Since the resulting material exhibited a time- and temperature-controlled viscosity, it can be a potential precursor for the production of other carbon based micro- and possibly, nano-sized gratings. Furthermore, the 2θ peak at 18° in the XRD spectra in Figure 6b, indicative of an amorphous aromatic carbon structure for the thermoset, shifted towards wider diffraction angles, coupled with the rise of a new 2θ band at 43°. It is noteworthy that both of these peaks become narrower as the aromatic planes begin to organize into glassy carbon upon exposure to higher temperatures [111].

In another study [54], a mixture of branched, processable oligomers (*M_n_* = 2500), and monomers was obtained through simple heat treatment of the neat monomer 2 in Figure 5b at 250 °C for 60 min in N_2_ gas. In the absence of solvents or additives, the resulting mixture exhibited low viscosity at 80 °C, making it useful for micromolding in capillaries (MIMIC), where the mixture was subsequently patterned with microhexagonal features imprinted from a PDMS mold and thermally cured. Upon a non-oxidative pyrolysis above 1000 °C, the final BODA thermoset showed high yields of dense monolithic carbon structures, accompanied by a 15% dimensional shrinkage (Figure 7b). In addition, the thermoset glass-like carbon product was lightweight, hard, conductive, chemically resistant, and gas-impermeable [114,115,116,117], and could find potential use in MEMs, NEMs, and other microfabricated electro-optical components [118,119,120,121,122,123]. BODA-derived hollow- (inner diameter of 400 µm) and cylinder-like (650 µm diameter) carbon surfaces were also fabricated.

ODA-based materials can also be used in the production of carbon fibers [58] and silica-templated photonic crystals [100]. Particularly, the BODA approach provides a more facile production of inverse opaline structures, relative to other polymeric carbon precursors. Using a phenolic resin, for example, would require the use of soft lithography and/or long pyrolysis times. On the other hand, the versatile BODA monomer structure containing terminal hydroxyl groups on the *ortho*-alkynes allows greater compatibility with the silica template, while the *ortho*-alkynes provide access to highly unsaturated organic residues capable of an atom-economic transformation to highly aromatic structures [100].

Furthermore, it has been found in several recent studies that the reactive didehydronaphthalene intermediate produced from the Bergman cyclization of ODA monomers can directly graft to a variety of carbonaceous surfaces including multi-walled fullerenes (carbon nano-onions, CNOs) [124], graphene [125], and multi-walled carbon nanotubes (MWCNTs) [126]. These ODA-based thermosets are modifiable by their spacer and terminal alkyne, allowing for specific functionalization for advanced applications [127,128]. Not only are the aryl diradicals extremely reactive, but the ODA aryldiradicals are stable, with long shelf life at room temperature [129]. Such materials can be practical, not only for fabricating monolithic carbon parts, but also for modifying carbon surfaces as they can be used to join carbon junctions and potentially treat carbon fiber surfaces in the same way the diazonium salts are used without the need for electrochemical reductions. Currently, our group is active in fabricating hierarchically reinforced carbon fiber composites based on these reactions and modifications.

## 4. Conclusions and Perspectives

The polymerization of BODA monomers by Bergman cycloaromatization produces highly reactive diradicals that react in a non-selective manner, leading to non-linear irregular structures. Due to their average functionality, these monomers are highly crosslinkable. Further, they can easily attain a high polydispersity, following a step-growth polymerization, and highly branched aromatic polymers that are solution and melt processable. It is noteworthy that their high carbon yield and low dimensional shrinkage make them suitable for large-scale fabrication of glass-like carbons, where other commercially available resins may fail to produce dense monolithic structures and require resin reinfusions to achieve optimal densities. Additionally, their ability to functionalize carbon surfaces has only been recently explored and will open new opportunities on the development of functional carbon materials.

Likewise, due to their unique combination of engineering thermoplastic (arylene ether polymer) and fluorocarbon attributes, semi-fluorinated PFCB materials have been widely explored for high performance surface coatings, membrane layers, dielectrics, optical materials, space survivability, fuel cell membranes, polymer composites and nanocomposites, and other more recent modular approaches. Their chemistry represents a new versatile cycloaddition step-growth type polymerization mechanism, the utility of which is currently under exploratory development at several research organizations including our group. Overall, PFCB and ODA-based polymers make up a unique class of atom-economic, ring-forming polymerization (which addresses the processability/performance trade-off issue), with ease of utility in the manufacture of advanced functional materials, requiring no additional reagents, solvents, or catalysts, and without producing waste or unwanted side-products.

## Data Availability

Not applicable.
